# Diffusion‐Weighted Imaging for the Evaluation of the Sacroiliac Joint in Pediatric Patients

**DOI:** 10.1002/acr.25661

**Published:** 2026-01-20

**Authors:** Michael L. Francavilla, Timothy G. Brandon, Dmitry Khrichenko, Rui Xiao, Nancy A. Chauvin, Asef Khwaja, Pamela F. Weiss

**Affiliations:** ^1^ Whiddon College of Medicine, University of South Alabama Mobile; ^2^ Children's Hospital of Philadelphia Philadelphia Pennsylvania; ^3^ Perelman School of Medicine University of Pennsylvania Philadelphia; ^4^ The Cleveland Clinic Cleveland Ohio

## Abstract

**Objective:**

Maturational signal in the sacroiliac joint (SIJ) of skeletally immature youth is often misinterpreted as inflammation. Diagnostic tools that improve specificity are greatly needed. Apparent diffusion coefficient (ADC) values from diffusion‐weighted imaging (DWI), when used with standard imaging, may enhance diagnostic accuracy. We aimed to define normative pediatric ADC values and establish thresholds to distinguish normal from inflammatory SIJ signals.

**Methods:**

ADC values were measured using circular regions of interest (ROIs) on the anterior, central, and posterior slices of the cartilaginous SIJs (36 total ROIs). Mean ADCs were analyzed by age group, bone (iliac or sacral), and joint height (superior, mid, inferior), accounting for within‐patient clustering. In sacroiliitis cases, ROIs were placed on DWI at sites of increased signal on fluid‐sensitive sequences. Thresholds differentiating normal and inflammatory signals were derived by age, bone, and joint height (ilium only) and assessed by area under the receiver operating characteristic (AUROC) and specificity.

**Results:**

The reference group included 86 youth. Inferior ilium ADC values were higher than mid and superior regions in all immature age groups (all *P* < 0.0001) and decreased with age (*P* = 0.0001). Sacral ADCs also declined with age (*P* = 0.0001). No age trend was observed in the superior or mid ilium (*P* = 0.14). ADC thresholds distinguished normal from inflammatory signals with AUROC ≥0.90 in most iliac regions, except the peripubertal inferior ilium (AUROC 0.78). Sacral thresholds performed acceptably (AUROC ≥ 0.77), though they were lower in the prepubertal group (AUROC 0.68).

**Conclusion:**

Age‐ and bone‐specific ADC reference values were established and effectively differentiated normal from inflammatory SIJ signals.

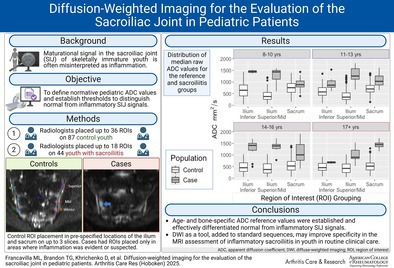

## INTRODUCTION

The moment sacroiliitis is detected in juvenile spondyloarthritis (enthesitis‐related arthritis [ERA] or psoriatic arthritis) is a key therapeutic decision point—axial disease does not respond to first‐line disease‐modifying agents such as methotrexate and warrants consideration of biologic medications.^1^ Up to one‐third of children with spondyloarthritis develop sacroiliitis within several years of diagnosis.^2^, ^3^, ^4^, ^5^ Magnetic resonance imaging (MRI) is the most used imaging modality to diagnose sacroiliitis in children. However, it has variable sensitivity and specificity in everyday practice and is reliant on the experience of the interpreting radiologist. Recent work across larger tertiary care centers in the US demonstrated that when leveraging dedicated pelvic MRI sequences and a central imaging team as a reference standard, sensitivity of local radiologists to identify sacroiliitis is high (>90%), but the specificity (median: 68%, interquartile range [IQR]: 53%–86%) and positive predictive values (median: 57%, IQR: 41%–57%) are moderate and low, respectively.^6^ The most common error is misinterpretation of the normal physiologic metaphyseal‐equivalent signal as an inflammatory signal.

**SIGNIFICANCE & INNOVATIONS**

- Apparent diffusion coefficient reference ranges were established by age group along the iliac and sacral sacroiliac joint.
- Cut‐offs to differentiate normal signal from pathologic inflammation had excellent and moderate area under the receiver operating characteristics at the ilium and sacrum, respectively.

In pediatric imaging, the signal of the metaphyseal‐equivalent part beneath the articular surface may mimic the inflamed bone marrow signal in children with unfused or partially fused sacroiliac apophyses; immature bone marrow has higher water content than mature bone marrow.^7^ There is no gold standard for the determination of normal versus pathologic inflammatory signal. As such, the reference standard for published studies is the consensus of musculoskeletal radiologists experienced in not only the diagnosis of sacroiliitis but also in the interpretation of the maturing sacroiliac joint (SIJ).^6^, ^8^, ^9^, ^10^, ^11^, ^12^ Diffusion‐weighted imaging (DWI) is a widely available MRI technique that reflects the diffusion of water within intra‐ and extra‐cellular tissue compartments. The apparent diffusion coefficient (ADC) is a quantitative assessment of directionally averaged water diffusion and can provide a quantitative measure of inflammation. Although not completely objective, ADC from DWI may facilitate interpretation. In adults, ADC has been shown to distinguish active from inactive sacroiliitis and can be followed over time to assess response to treatment.^13^, ^14^, ^15^, ^16^, ^17^ Small studies in children have confirmed similar findings.^10^, ^18^, ^19^ In adults, the addition of DWI to standard fluid‐sensitive sequences significantly enhanced the specificity of the imaging assessment from 67% to 80%, although the authors concluded the addition of DWI did not improve overall accuracy, sensitivity, or confidence in the ascertainment of sacroiliitis.^20^ In pediatrics, the issue is not failure to detect sacroiliitis; it is primarily misinterpretation of normal maturational signals for inflammation, so tools that can provide incremental improvements in specificity, not sensitivity, are greatly needed. We hypothesize that ADC from DWI may be a useful tool to improve the specificity in the interpretation of the pediatric SIJ imaging evaluation, in addition to standard sequences, while maintaining the sensitivity. This project aimed to define normative ADC values in a pediatric reference population and establish data‐driven thresholds to distinguish normal from inflammatory SIJ signal.

## PATIENTS AND METHODS

This was a retrospective cross‐sectional study consisting of two phases. In phase 1, standards of ADC interpretation in a reference population of control patients were generated, and in phase 2, empirical data‐driven thresholds to differentiate normal and inflammatory signals were developed.

### Ethics

This study met criteria for exemption and was granted a waiver of informed consent by the Children's Hospital of Philadelphia Institutional Review Board (20‐018172).

### Patients

#### Reference control group

The reference control group included 87 youth. Twenty‐nine participants were prospectively recruited from a primary care practice as part of a prior imaging study; they had no personal history of back pain, trauma, surgery, or known endocrine, oncologic, or rheumatologic disease.^21^ An additional 58 patients were retrospectively identified through a query of the picture archiving and communication system (PACS). These individuals underwent standard MRI, including DWI, as part of routine clinical evaluation for concerns such as stress fractures, infection, or mechanical back pain—but none were ultimately found to have underlying pathology. All imaging studies were rereviewed by a central review team to confirm the absence of abnormalities, ensuring their suitability as controls.

#### Established sacroiliitis group

The sacroiliitis group included 44 patients. Thirty‐four were prospectively recruited from rheumatology clinics at three centers as part of a separate study.^22^ All had a clinical diagnosis of sacroiliitis and met criteria for ERA or psoriatic arthritis based on the International League of Associations for Rheumatology (ILAR)^23^ or European Spondyloarthropathy Study Group (ESSG)^24^ classification criteria. An additional 10 patients with ERA and sacroiliitis were identified retrospectively through a PACS query. In all cases, sacroiliitis was confirmed by clinical assessment along with standard MRI, demonstrating periarticular bone marrow edema‐like signal. Demographic information was obtained directly from participants or extracted from the electronic medical record.

### Imaging protocol

Oblique coronal small field‐of‐view T1‐weighted, fluid‐sensitive STIR sequences and axial DWI sequences of the SIJs were acquired according to either (a) the research study protocol in which the patient enrolled in or (b) the institutional pelvic imaging protocol used for clinical care. Both protocols used three‐directional DWI with single‐shot spin‐echo echo‐planar imaging. Diffusion gradient b values varied, with most sequences using 50, 400/500, and 800/1000 s/mm^2^. All scans (N = 123) were performed at 3.0 Tesla. MRI manufacturers and models are detailed in Supplementary Tables 1 and 2. Slice thicknesses for T1, STIR, and DWI sequences ranged from 3 to 5 mm.

### Imaging interpretation

#### Reference control group

The DWI and STIR images were anonymized and reviewed independently by two experienced pediatric musculoskeletal radiologists using custom software (Parametric MRI; https://www.parametricmri.com/). Both radiologists had expertise in imaging evaluation of sacroiliitis and interpreting imaging of the maturing SIJ. They completed real‐time iterative calibration modules to optimize scoring accuracy of SIJ MRI lesions.^25^, ^26^ Axial DWI sequences were reformatted by the software into a semicoronal plane to facilitate comparison with fluid‐sensitive STIR sequences and placement of regions of interest (ROIs).

**Figure 1:**
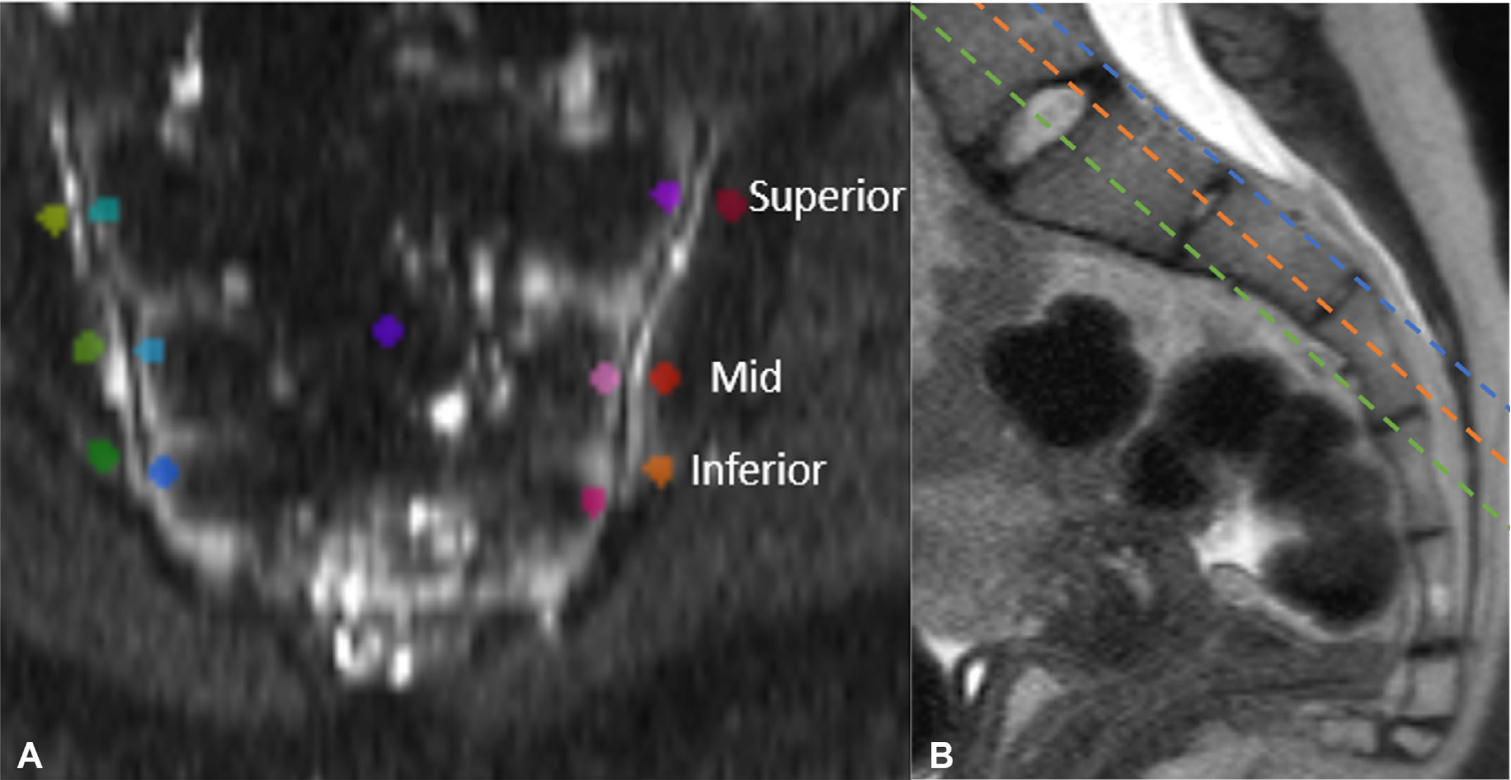
ROI placement on control patients. (a) Sample placement of ROIs in the SIJ on the central slice of the coronal oblique imaging plane. Analogous ROI placement was performed on the SIJs in one anterior slice and one posterior slice. (b) Sagittal view of the SIJ showing the anterior (orange), central (green), and posterior (blue) SIJ acquisition planes. ROI, region of interest; SIJ, sacroiliac joint.

Interpretation standards, terminology, and ROI placements were established through a consensus review of 10 reference control cases. To define normative ADC reference ranges, up to 12 circular ROIs were placed per anterior, central, and posterior slice of the SIJ in each patient, following a method similar to Bozgeyik et al.^13^ On each slice, ROIs were placed on both iliac and sacral sides along the cartilaginous SIJ, divided into superior, mid, and inferior portions of the joint (Figure 1). The central coronal slice was selected to include the S1 and S2 vertebral bodies, choosing the slice showing the greatest portion of both if multiple slices qualified. ROIs were omitted if part of the joint was not visible. Each control patient had up to 36 ROIs evaluated.

ROI volumes ranged from 0.15 to 0.22 cm^2^ and were placed on subchondral bone marrow, including any apophyseal growth cartilage but excluding the articular bone cortex. All reference control studies were evaluated with the protocol for ROI placement described in the Imaging Protocol section. ADC values were calculated by the software using a best‐fit linear logarithmic regression of all b values.

#### Established sacroiliitis group

For patients with confirmed sacroiliitis, ROIs were identified through two distinct exercises. First, two radiologists independently placed ROIs on areas demonstrating high signal intensity on STIR images, simulating how ADC might be applied in routine clinical practice. Second, the central review team conducted a consensus session to collaboratively place ROIs across all cases. This second step aimed to optimize placement for establishing diagnostic thresholds. The sequence of these exercises was intentionally designed to reduce bias during the independent ROI placement.

**Figure 2:**
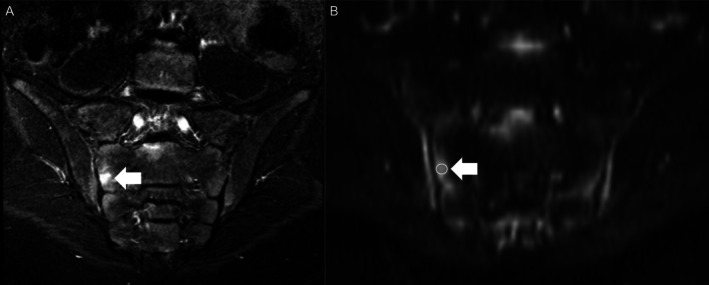
ROI placement on patients with established sacroiliitis. ROI, region of interest.

In both exercises, subchondral bone marrow and apophyseal cartilage were evaluated for signal intensity and appearance on coronal oblique fluid‐sensitive STIR images, along with corresponding signal on the ADC map. ROIs were placed in regions showing increased signal (see Figure 2). Similar to ROI placement in the reference control group, care was taken to avoid areas of erosion, adjacent cortical bone, sclerosis, or fat metaplasia to prevent volume averaging and inaccurate ADC values. For patients with sacroiliitis, multiple ROIs were allowed to capture a representative range of signal intensities. However, placement was limited to one ROI each in the superior, mid, and inferior regions on the right and left iliac and sacral bones for every anterior, central, and posterior slice of the SIJs. The number of ROIs per case ranged from 1 to 18. Sample case in which edema signal was present in the central SIJ imaging plane on the (a) STIR image and (b) the corresponding placement of the ROI on the DWI sequence.

### Statistical analysis

Demographics and clinical characteristics were summarized using medians with IQRs or counts with percentages.

#### Phase 1: Reference limit estimation and group comparisons

Scatter plots and histograms were created to visually inspect the data. Reference limits (2.5th and 97.5th percentiles) were estimated using the mean ADC from both central reviewers for each ROI by age group (prepubertal: 8–10 years, peripubertal: 11–13 years, postpubertal: 14–16 years, and mature: ≥17 years), bone (iliac or sacral), and joint height (superior, mid, inferior), accounting for within‐patient clustering. Interrater agreement for all reference control studies was visualized using Bland–Altman plots, with limits of agreement defined as the mean difference ±1.96 SDs. Differences among ADC values across the inferior, mid, and superior regions of the anterior, central, and posterior slices of the iliac and sacral bones were assessed using the Kruskal–Wallis tests; Dunn's test was performed as a post‐hoc procedure following rejection of a Kruskal–Wallis test. A test for trend evaluated changes in ADC values with increasing age.

#### Phase 2: Threshold selection and performance

Thresholds differentiating normal and inflammatory signals were generated by age, bone, and joint height (ilium only) using the Liu method.^27^ This method optimizes the threshold for a diagnostic test using the product of the sensitivity and specificity. Each threshold was assessed by area under the receiver operating characteristic (AUROC), specificity, and sensitivity implemented in an R package “fastAUC.”^28^ AUROC of less than 0.60 was considered poor; 0.60 to 0.75, moderate; and more than 0.75, excellent discrimination.^29^, ^30^ Analyses were conducted using Stata version 17 (StataCorp LLC) and R version 4.4.1.^31^

## RESULTS

A total of 87 youth were included in the reference group to develop normative ADC values, whereas an additional 44 youth with confirmed sacroiliitis were used to establish data‐driven thresholds for distinguishing normal from inflammatory SIJ signal. The reference control group consisted of youth in the 8 to 10 (n = 23), 11 to 13 (n = 19), 14 to 16 (n = 27), and 17+ (n = 18) years age groups. In comparison to the control population, there were fewer patients who were female (36.4% vs 43.7%), ages 8 to 10 years old (13.6% vs 26.4%), and 17 years old or older (15.9% vs 20.7%) and more patients who were 11 to 13 years old (34.1% vs 21.8%) and 14 to 16 years old (36.4% vs 31.0%), but those differences were not statistically significant.

### Establishment of normative ADC values and evaluation of age‐related trends

Supplementary Table 3 shows the range and median (IQR) of ADC values in the reference population, grouped by age, bone (iliac or sacral), and joint height (superior, mid, or inferior). In the ilium, ADC values at the superior and mid joint heights did not differ significantly within age groups and were therefore combined. In contrast, the inferior ilium showed significantly higher ADC values than the mid or superior region in the pre‐, peri‐, and post‐pubertal age groups (age < 17 years, all *P* < 0.0001). ADC in the inferior ilium decreased significantly with age (*P* = 0.0001), whereas no significant age‐related trend was observed in the combined superior and mid regions (*P* = 0.14). In the sacrum, ADC values did not differ significantly by joint height (superior, mid, inferior), but overall sacral ADC values declined significantly with age (*P* = 0.0001).

### Interobserver agreement

Interrater agreement, as assessed by a Bland–Altman plot, is shown in the Supplementary Figure 1. The mean difference in ADC assessments between raters was −36.2 units, which was relatively close to zero given the range of values, suggesting no significant bias between the two raters. The 95% limit of agreement was −719 to 647. The heteroscedasticity or spread of points increased with average rater ADC values, with a variance for the ROIs with an average rater ADC greater than 800 (18.5% of the ROI data points) over 2.5 times that of the variance for ROIs with an average rater ADC less than 800, indicating less reliable agreement at extreme values.

### Threshold selection

**Figure 3:**
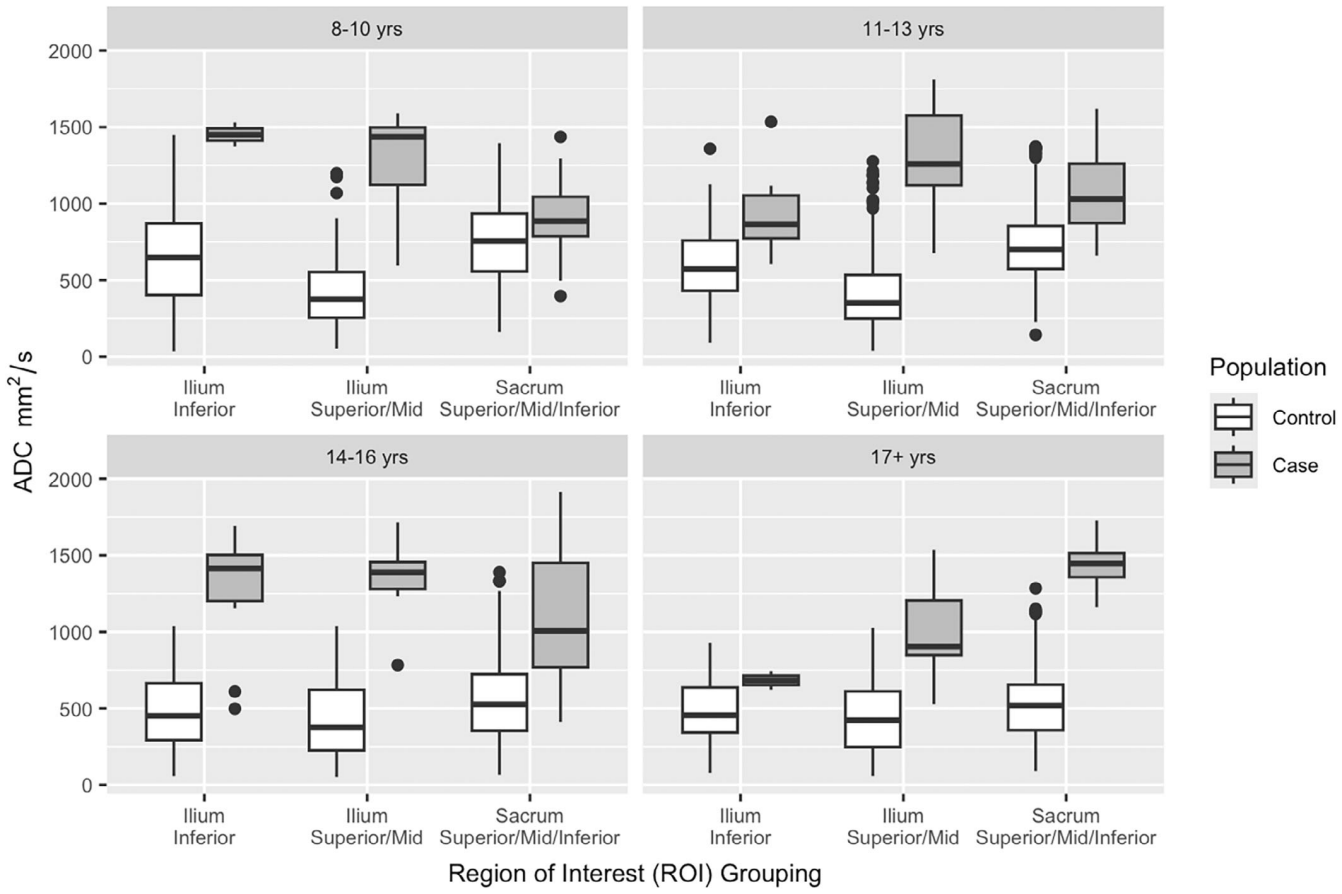
Boxplots of ADC values of controls and patients with sacroiliitis by ROI groupings. ADC, apparent diffusion coefficient; ROI, region of interest.

Figure 3 shows the distribution of median raw ADC values for the reference and sacroiliitis groups. Table 1 presents empirically derived thresholds for distinguishing normal maturational signals from inflammatory signals, along with their corresponding AUROC, specificity, and sensitivity in two populations: (1) controls plus consensus ROI cases and (2) controls plus the average independent ROI placement cases (performance of the independent rater ROI placements on cases is available in Supplementary Table 4). These thresholds were established using normative values from phase 1 and consensus ROI placement in the sacroiliitis cases.

**Table 1:** Empiric thresholds and test properties to differentiate normal from inflammatory signal*

ROI location	Age group, y	n^a^	Cut point (95% CI)	Case ROI source	AUROC	Specificity	Sensitivity
Ilium							
Superior/Mid	8–10	260	854 (601–1,271)	Consensus	0.91 (0.84–0.99)	0.97 (0.95–0.99)	0.84 (0.69–1)
				Independent	0.84 (0.63–0.93)	0.99 (0.97–1)	0.69 (0.45–0.93)
Inferior	8–10	86	1,338 (1,332–1,489)	Consensus	0.98 (0.95–1)	0.8 (0.66–0.95)	0.56 (0.19–0.92)
				Independent	0.68 (0.52–0.91)	0.95 (0.88–1.03)	0.4 (0.09–0.71)
Superior/Mid	11–13	222	849 (678–1,195)	Consensus	0.93 (0.84–0.99)	0.92 (0.84–1)	0.95 (0.84–1.05)
				Independent	0.9 (0.83–0.96)	0.93 (0.86–1.01)	0.87 (0.77–0.98)
Inferior	11–13	100	794 (593–967)	Consensus	0.78 (0.62–0.9)	0.78 (0.68–0.88)	0.79 (0.66–0.91)
				Independent	0.72 (0.55–0.86)	0.76 (0.64–0.88)	0.68 (0.4–0.96)
Superior/Mid	14–16	267	772 (764–1,211)	Consensus	0.94 (0.9–0.98)	0.94 (0.87–1.01)	0.88 (0.71–1.05)
				Independent	0.84 (0.74–0.94)	0.89 (0.82–0.97)	0.79 (0.6–0.98)
Inferior	14–16	129	1,097 (1,072–1,272)	Consensus	0.9 (0.5–1)	0.77 (0.65–0.9)	0.77 (0.57–0.97)
				Independent	0.77 (0.5–0.88)	0.96 (0.92–1)	0.57 (0.29–0.86)
Superior/Mid	17+	216	839 (836–980)	Consensus	0.91 (0.79–0.98)	0.94 (0.9–0.99)	0.87 (0.77–0.98)
				Independent	0.87 (0.7–0.95)	0.96 (0.92–1)	0.79 (0.57–1)
Inferior	17+	82	613 (610–1,269)	Consensus	0.86 (0.78–0.93)	0.88 (0.76–0.99)	0.74 (0.53–0.95)
				Independent	0.81 (0.6–0.91)	0.7 (0.52–0.88)	0.92 (0.74–1.1)
Sacrum							
Superior/Mid/Inferior	8–10	342	767 (763–890)	Consensus	0.68 (0.57–0.76)	0.52 (0.41–0.62)	0.83 (0.73–0.94)
				Independent	0.69 (0.62–0.78)	0.52 (0.42–0.63)	0.86 (0.76–0.96)
Superior/Mid/Inferior	11–13	299	860 (788–1,136)	Consensus	0.78 (0.66–0.87)	0.76 (0.68–0.83)	0.81 (0.64–0.99)
				Independent	0.71 (0.64–0.8)	0.73 (0.65–0.8)	0.69 (0.54–0.84)
Superior/Mid/Inferior	14–16	435	741 (609–969)	Consensus	0.77 (0.63–0.88)	0.76 (0.67–0.85)	0.79 (0.56–1.02)
				Independent	0.7 (0.58–0.79)	0.76 (0.68–0.85)	0.64 (0.46–0.82)
Superior/Mid/Inferior	17+	295	1,156 (1,150–1,332)	Consensus	1 (0.99–1)	0.79 (0.64–0.95)	0.8 (0.44–1.16)
				Independent	0.81 (0.65–0.93)	0.79 (0.64–0.95)	0.8 (0.44–1.16)

* AUROC of less than 0.60 was considered poor discrimination, 0.60 to 0.75 moderate discrimination, and more than 0.75 excellent discrimination. AUROC, area under the receiver operating characteristic; CI, confidence interval; ROI, region of interest.

^a^ n = ROIs assessed at each location per maturational group.

For the ilium, thresholds achieved AUROC values ≥0.90 across all age groups (pre‐, peri‐, and post‐pubertal) in both the superior/mid and inferior regions—except in the peripubertal group, in which the inferior ilium had a lower AUROC of 0.78. Specificity was ≥0.92 for the superior and mid ilium and ≥0.77 in the inferior ilium across all age groups. In the sacrum, AUROC and specificity were ≥0.77 and ≥0.76, respectively, for all age groups—except in the prepubertal group, in which AUROC dropped to 0.68 and specificity to 0.52.

**Figure 4:**
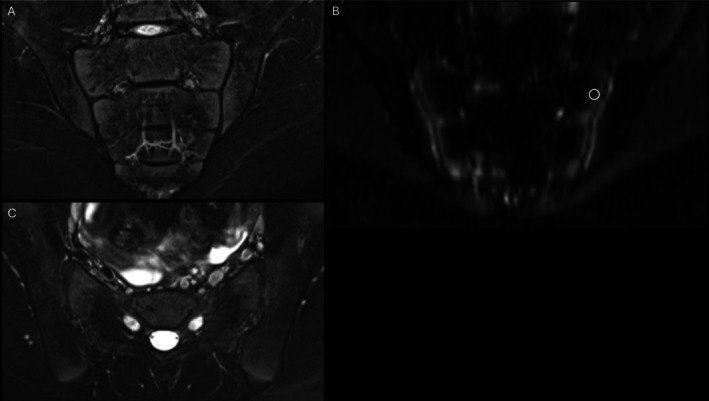
Representative application of the DWI technique. (A) Coronal oblique STIR showing normal type I maturational signal. (B) Coronal oblique DWI showing ROI placement in the superior left sacrum. Measured ADC was 666 mm^2^/s. (C) Axial oblique T2 fat‐saturated image showing normal maturational signal in the iliac and sacral bones about the SIJs and iliac apophyses. ADC, apparent diffusion coefficient; DWI, diffusion‐weighted imaging; ROI, region of interest; SIJ, sacroiliac joint.

When thresholds were applied to data from an independent ROI placement exercise (Figure 4)—in which each reviewer independently placed ROIs in areas of high STIR signal to simulate real‐world clinical application—the superior and mid ilium showed AUROC values ≥0.84 across all age groups. In the inferior ilium, AUROC values were ≥0.72 across all age groups, except in the prepubertal group, in which the AUROC was 0.68. Notably, specificity values for the ADC thresholds in the ilium in all age groups were equal to or higher than those obtained from the consensus ROI exercise.

## DISCUSSION

Differentiating inflammatory sacroiliitis from its mimics based on symptoms and clinical examination alone is challenging. As a result, MRI findings increasingly guide treatment decisions. However, interpreting sacroiliac imaging in skeletally immature patients remains difficult,^6^, ^11^, ^12^, ^21^, ^32^, ^33^, ^34^ and there is a need to avoid unnecessary use of biologics, which carry significant risks and costs. Therefore, additional tools that enhance imaging accuracy—that are simple, accessible, and complementary to standard sequences—are needed. This study achieved two key goals. First, it established normative age‐ and bone‐specific ADC values in the SIJs of youth, revealing that the inferior ilium and sacral ADCs decline with age, whereas superior and mid ilium values remain stable. Second, it derived empirical thresholds to differentiate normal from inflammatory signals, demonstrating strong performance (AUROC ≥0.90 in most iliac regions and ≥0.77 in most sacral regions). A recent multicenter study found, with dedicated MRI sequences, that radiologists at large tertiary care academic pediatric hospitals achieved high sensitivity (>90%) for detecting sacroiliitis but only moderate specificity (median 68%).^6^ The current findings suggest that the ADC from DWI sequences may meaningfully enhance the specificity and overall accuracy of MRI evaluation for inflammatory sacroiliitis, particularly in the pediatric population.

A growing body of evidence supports the use of DWI sequences for assessing sacroiliitis in both adults^13^, ^15^, ^35^ and children.^9^, ^10^, ^19^ DWI is most commonly applied to highly vascularized tissues or those with high water content, such as the brain. At the SIJ, DWI may be useful in detecting inflammation by identifying increased water content in the subchondral bone marrow. However, in adults, it remains debated whether quantitative measures from DWI—specifically ADC values—provide added diagnostic value, especially in cases in which inflammation is subtle and the signal may be difficult to accurately quantify.^36^ In pediatric patients, the challenge is different. Maturational bone marrow signal is often pronounced and can easily mimic pathology.^6^, ^11^, ^12^, ^21^, ^32^, ^33^, ^34^ Unlike adults with longstanding axial disease, who frequently exhibit structural lesions that complicate ROI placements and ADC interpretation, children are typically evaluated early in the disease course—often before structural changes are detectable. This presents a unique opportunity to leverage DWI more effectively in the pediatric setting, specifically, to aid in improved specificity and overall accuracy of MRI evaluation.

Several studies have explored the use of DWI sequences to evaluate sacroiliitis.^9^, ^13^, ^18^, ^20^, ^35^, ^37^ We leveraged key insights from these studies to inform the design of our ROI placement strategy, including the number, size, and positioning. Coronal oblique sequences were selected for ROI placement rather than axial sequences, as they provide better visualization of the SIJ and facilitate more accurate ROI positioning. Vendhan et al used the mid‐sacrum to normalize the ADC values.^9^ However, their study showed that normalized ADC values of patients with mild inflammation were similar to those in controls with immature SIJs. Additionally, the variability in reference ADC values contributed substantially to the spread of normalized results. Normalization also reduced the intraclass correlation coefficient and widened the Bland–Altman limits of agreement, suggesting that the use of uncorrected ADC values might yield better interobserver reproducibility than normalized ones. These findings indicate that normalizing ADC may not be ideal when attempting to define empirical ADC thresholds.^9^ For this reason, we chose not to normalize our ADC data to the mid‐sacrum.

Previous DWI studies have several limitations, including not accounting for age or sex differences,^38^ placing ROIs across the joint instead of within bone,^9^, ^18^ using few ROIs, and introducing subjectivity in ROI placement. This study addresses age‐related variation by stratifying results by age, though it was underpowered to assess sex differences. Although ROI placement on areas of edema remains somewhat subjective, standardization was prioritized—defining the central coronal slice, setting subchondral ROI criteria, and providing reproducible guidance for less experienced users. Circular ROIs were used to ensure clinical feasibility, as they can be generated in standard PACS systems. Larger ROIs were selected to capture a range of signals and better define cut‐off values. Although custom semi‐automated software offers more precision, it is not widely accessible. Our software also adjusts for differences in MRI scanners (manufacture and model), imaging protocols, and ADC calculations^39^ across institutions, improving generalizability. Reference values are tied to joint position, allowing flexibility in image acquisition. If axial images are obtained, our program can reformat them to a semicoronal plane—though at the cost of some spatial resolution.

The relatively wide Bland–Altman limits and high variability in bone ADC due to low signal and noise sensitivity could be considered limitations. Signal‐poor structures like bone have an expected high amount of variance due to noise. Therefore, small changes in ROI placement result in substantial variation in measured mean ADC. However, the differences in mean ADC between controls and cases were generally large, reflecting the power of DWI to differentiate between noise in controls and abnormal signal in cases. Although ROI selection was based on STIR images—introducing potential bias—interpreters were experienced and well calibrated. The sample size was modest, but AUROC values for derived thresholds were strong. This study did not assess the fat content within the bone marrow, which may influence ADC values,^40^ or the additive diagnostic value of DWI. This study also did not assess the impact of physical activity on ADC, as physical activity was not recorded for the reference control group or the patients with sacroiliitis. The reference control group also underwent imaging for a range of clinical reasons. However, the intent of this tool is to be applied to children undergoing evaluation for back pain stemming from diverse potential causes. In this context, the heterogeneity of our control group is actually a strength, as it enhances the real‐world clinical relevance and applicability of the tool. Lastly, variability in pediatric SIJ interpretation is widespread^6^ and multifactorial. Although no single solution exists, DWI and improved training and/or automated image analysis may enhance diagnostic accuracy.

In summary, this study establishes normative age‐ and bone‐specific ADC values for the pediatric SIJ and demonstrates that ADC thresholds from DWI sequences can distinguish normal maturation from inflammatory signal. By addressing limitations of prior studies—such as variation by age, ROI placement inconsistency, and normalization issues—this work supports the use of DWI as a potential practical tool to improve specificity in the MRI assessment of inflammatory sacroiliitis in youth. DWI is not intended to replace standard imaging; rather, it may offer meaningful incremental value, particularly given the unique challenges of interpreting skeletally immature joints and the clinical importance of avoiding unnecessary biologic treatment. Future research should further evaluate the impact of marrow fat content, assess diagnostic value in conjunction with standard sequences, and explore automated approaches to enhance reproducibility and diagnostic accuracy.

In summary, this study established normative age‐ and bone‐specific ADC reference ranges for the pediatric SIJ and demonstrated that ADC thresholds from DWI can distinguish normal maturational signal from inflammatory signal with high AUROC and specificity. This work supports the continued exploration of DWI as a tool, in addition to standard sequences, to improve specificity in the MRI assessment of inflammatory sacroiliitis in youth in routine clinical care. Future work should further evaluate the impact of marrow fat content, assess diagnostic value in conjunction with standard sequences, and explore automated approaches to enhance reproducibility and diagnostic accuracy.

## AUTHOR CONTRIBUTIONS

All authors contributed to at least one of the following manuscript preparation roles: conceptualization AND/OR methodology, software, investigation, formal analysis, data curation, visualization, and validation AND drafting or reviewing/editing the final draft. As corresponding author, Dr Francavilla confirms that all authors have provided the final approval of the version to be published and takes responsibility for the affirmations regarding article submission (eg, not under consideration by another journal), the integrity of the data presented, and the statements regarding compliance with institutional review board/Declaration of Helsinki requirements.

## Supporting information


**Disclosure form**.

**Appendix S1:** Supplementary Information.
