# Supplementary material for: Diffusion‐Weighted Imaging for the Evaluation of the Sacroiliac Joint in Pediatric Patients
Source: Arthritis Care Res (Hoboken). 2026 Jan 20;78(5):630–8. doi: 10.1002/acr.25661 (PMC13116032; doi:10.1002/acr.25661)
Supplement: Supplementary file 2 — Appendix S1: Supplementary Information. [file ACR-78-630-s001.docx]

**Supplement Table 1.** MRI (3.0T) scanners used to collect diffusion weighted imaging sequences.


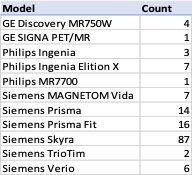


Counts include subjects who were excluded due to image quality

**Supplement Table 2.** Protocol specifications for STIR sequences on 3.0T MRI

|  | Time of repetition (TR) | Time of echo (TE) | Time of inversion (TI) |
| --- | --- | --- | --- |
| Mean | 4736.8 | 46.7 | 210.7 |
| Range | 3000-9440 | 31-97 | 180-230 |

**Supplement Table 3.** Control population apparent diffusion coefficients in the sacroiliac joints.

| **Age Group** | **ROI Location** | **N*** | **Range** | **Median (IQR)** |
| --- | --- | --- | --- | --- |
| **Ilium** | | | | |
| 8-10 years | Superior | 116 | 54-877 | 443 (291-616) |
|  | Mid | 123 | 60-835 | 331 (206-481) |
|  | Inferior | 83 | 122-1661 | 670 (433-922) |
| 11-13 years | Superior | 93 | 82-1133 | 339 (251-507) |
|  | Mid | 105 | 51-1184 | 359 (242-539) |
|  | Inferior | 87 | 110-1357 | 580 (433-793) |
| 14-16 years | Superior | 128 | 48-1025 | 387 (192-638) |
|  | Mid | 134 | 39-975 | 356 (231-577) |
|  | Inferior | 127 | 57-1393 | 498 (293-721) |
| ≥17 years | Superior | 97 | 57-901 | 428 (250-565) |
|  | Mid | 100 | 57-911 | 410 (247-604) |
|  | Inferior | 80 | 88-1295 | 469 (348-642) |
| **Sacrum** | | | | |
| 8-10 years | Superior | 118 | 161-1350 | 782 (599-953) |
|  | Mid | 127 | 205-1368 | 763 (562-929) |
|  | Inferior | 82 | 174-1311 | 668 (518-858) |
| 11-13 years | Superior | 96 | 341-1372 | 738 (603-928) |
|  | Mid | 105 | 349-1516 | 695 (586-892) |
|  | Inferior | 83 | 331-1467 | 737 (574-851) |
| 14-16 years | Superior | 131 | 103-1266 | 546 (343-740) |
|  | Mid | 143 | 112-1330 | 527 (348-777) |
|  | Inferior | 128 | 107-1237 | 521 (371-697) |
| ≥17 years | Superior | 101 | 39-1017 | 518 (313-609) |
|  | Mid | 98 | 35-1128 | 522 (336-675) |
|  | Inferior | 84 | 58-1092 | 484 (313-618) |

*N=ROI assessed at each location per age group. ROI=region of interest; IQR=interquartile range.

**Supplement Figure 1. Bland-Altman plot of Rater 1 vs. Rater 2 apparent diffusion coefficient (ADC) values in the control population.** The gray box represents the 95% agreement limit. ADC = Apparent diffusion coefficient

**Supplement Table 4.** **Empiric thresholds and test properties to differentiate normal from inflammatory signal.**

| **ROI Location** | **Age group** | **Cutpoint** | **Case ROI source** | **AUROC** | **Specificity** | **Sensitivity** |
| --- | --- | --- | --- | --- | --- | --- |
| **Ilium** | | | | | | |
| Superior/Mid | 8-10 yrs | 854 (601-1271) | Rater 1 | 0.8 (0.56-0.93) | 0.99 (0.97-1) | 0.61 (0.26-0.95) |
|  |  |  | Rater 2 | 0.88 (0.79-0.95) | 0.99 (0.97-1) | 0.77 (0.65-0.89) |
| Inferior | 8-10 yrs | 1338 (1332-1489) | Rater 1 | 0.6 (0.45-0.83) | 0.95 (0.88-1.03) | 0.25 (-0.14-0.64) |
|  |  |  | Rater 2 | 0.73 (0.54-0.97) | 0.95 (0.88-1.03) | 0.5 (0.15-0.85) |
| Superior/Mid | 11-13 yrs | 849 (678-1195) | Rater 1 | 0.88 (0.78-0.96) | 0.93 (0.86-1.01) | 0.82 (0.67-0.98) |
|  |  |  | Rater 2 | 0.94 (0.87-0.99) | 0.93 (0.86-1.01) | 0.95 (0.87-1.03) |
| Inferior | 11-13 yrs | 794 (593-967) | Rater 1 | 0.68 (0.51-0.86) | 0.76 (0.64-0.88) | 0.61 (0.31-0.9) |
|  |  |  | Rater 2 | 0.68 (0.47-0.87) | 0.76 (0.64-0.88) | 0.61 (0.2-1.01) |
| Superior/Mid | 14-16 yrs | 772 (764-1211) | Rater 1 | 0.81 (0.69-0.94) | 0.89 (0.82-0.97) | 0.73 (0.51-0.95) |
|  |  |  | Rater 2 | 0.86 (0.68-0.95) | 0.89 (0.82-0.97) | 0.82 (0.62-1.02) |
| Inferior | 14-16 yrs | 1097 (1072-1272) | Rater 1 | 0.73 (0.49-0.98) | 0.96 (0.92-1) | 0.5 (0.15-0.85) |
|  |  |  | Rater 2 | 0.81 (0.54-0.98) | 0.96 (0.92-1) | 0.67 (0.3-1.04) |
| Superior/Mid | 17+ yrs | 839 (836-980) | Rater 1 | 0.82 (0.68-0.93) | 0.96 (0.92-1) | 0.69 (0.45-0.93) |
|  |  |  | Rater 2 | 0.95 (0.47-0.99) | 0.96 (0.92-1) | 0.95 (0.82-1.07) |
| Inferior | 17+ yrs | 613 (610-1269) | Rater 1 | 0.78 (0.38-0.91) | 0.7 (0.52-0.88) | 0.86 (0.57-1.15) |
|  |  |  | Rater 2 | 0.85 (0.76-0.93) | 0.88 (0.76-0.99) | 0.74 (0.53-0.95) |
| **Sacrum** | | | | | | |
| Superior/Mid/Inferior | 8-10 yrs | 767 (763-890) | Rater 1 | 0.69 (0.5-0.77) | 0.52 (0.42-0.63) | 0.86 (0.73-0.98) |
|  |  |  | Rater 2 | 0.67 (0.55-0.79) | 0.52 (0.42-0.63) | 0.82 (0.59-1.05) |
| Superior/Mid/Inferior | 11-13 yrs | 860 (788-1136) | Rater 1 | 0.71 (0.64-0.79) | 0.73 (0.65-0.8) | 0.69 (0.58-0.8) |
|  |  |  | Rater 2 | 0.7 (0.61-0.79) | 0.73 (0.65-0.8) | 0.68 (0.51-0.84) |
| Superior/Mid/Inferior | 14-16 yrs | 741 (609-969) | Rater 1 | 0.7 (0.58-0.78) | 0.76 (0.68-0.85) | 0.64 (0.46-0.82) |
|  |  |  | Rater 2 | 0.72 (0.57-0.81) | 0.76 (0.68-0.85) | 0.68 (0.46-0.89) |
| Superior/Mid/Inferior | 17+ yrs | 1156 (1150-1332) | Rater 1 | 0.8 (0.53-0.92) | 0.79 (0.64-0.95) | 0.8 (0.44-1.16) |
|  |  |  | Rater 2 | 0.87 (0.68-1) | 0.79 (0.64-0.95) | 0.8 (0.44-1.16) |
